# An Electronic Wellness Program to Improve Diet and Exercise in College Students: A Pilot Study

**DOI:** 10.2196/resprot.4855

**Published:** 2016-02-29

**Authors:** Amy L Schweitzer, Jamisha T Ross, Catherine J Klein, Kai Y Lei, Eleanor R Mackey

**Affiliations:** ^1^ Children's National Health System Clinical Research Center Washington, DC United States; ^2^ Department of Nutrition and Food Science University of Maryland College Park, MD United States; ^3^ Howard Community College Department of Health Sciences Columbia, MD United States; ^4^ Children's National Health System Department of Psychology and Behavioral Health Washington, DC United States

**Keywords:** college students, eHealth, telemedicine, diet, intervention studies, obesity, exercise

## Abstract

**Background:**

In transitioning from adolescence to adulthood, college students are faced with significant challenges to their health habits. Independence, stress, and perceived lack of time by college students have been known to result in poor eating and exercise habits, which can lead to increased disease risk.

**Objective:**

To assess the feasibility and to determine preliminary efficacy of an electronic wellness program in improving diet and physical activity in college students.

**Methods:**

A 24-week diet and physical activity program was delivered via email to 148 college students. The intervention involved weekly, tailored, and interactive diet and physical activity goals. The control group received nondiet and nonexercise-related health fact sheets. Anthropometric and blood pressure measurements, as well as food frequency and physical activity surveys were conducted at baseline, week 12, and week 24. Students’ choice of fruit as a snack was also monitored at study visits.

**Results:**

Students were 18-20 years old, 69% female, and from a diverse college campus (46% Caucasian, 23% Asian, 20% African American, 11% other). At week 24, 84% of students reported reading at least half of all emails. Mean change (standard error [SE]) from baseline of saturated fat intake was marginally significant between the treatment groups at week 24, 0.7 (SE 0.42) % kcal for control and -0.3 (SE 0.30) % kcal for intervention (*P*=0.048). A significant difference in percent of snacks chosen that were fruit (χ^2^_1_, N=221 = 11.7, *P*<0.001) was detected between the intervention and control group at week 24.

**Conclusions:**

Use of an electronic wellness program is feasible in college students and resulted in a decrease in saturated fat intake and an increase in observed fruit intake compared to a control group.

## Introduction

The phrase “freshman 15” was coined to describe weight gain associated with poor health habits in US college students. Studies of this phenomenon have identified an average of a 1-4 kg weight gain during the first year of college [1-4]. In the United States, this can result in increased proportions of students being classified as overweight, from 21% at the beginning of freshman year to 32% at the end of the first school year [1]. This weight gain can lead to obesity in adulthood. Specifically, the risk of being obese as an adult is 4 times as high for an overweight or obese adolescent, compared to a normal-weight adolescent [5]. Obese adolescents are at increased risk for disease as an adult, particularly cardiovascular disease [6-8].

College is a time of increasing independence and growth. During this period, students may make poor diet choices such as skipping breakfast, consuming salty and sugary snacks, and increasing alcohol consumption, and may fail to meet physical activity guidelines [1,4,9-13]. According to 2013 data from the National College Health Assessment, only 6% of college students report eating at least 5 servings of fruits and vegetables daily and less than half meet recommendations for exercise [14]. Such health behaviors result in a generation entering adulthood at risk for obesity, cardiovascular disease, and cancer. Clearly, there is a need to improve diet and exercise habits of college students.

Contemporary college students are a technologically wired generation having been born and raised in the age of home computers and portable electronic devices. Electronic health interventions (eHealth) have the advantage of reaching larger numbers of individuals with fewer resources than face-to-face interventions [15]. The duration of eHealth studies in college students has ranged from 30 days [16] to 2 years [17], with most studies reporting a duration of 12 weeks [18-22]. A variety of eHealth methods including virtual technology, graded classroom assignments, and Web-based coaching using Facebook, email, and SMS text messaging, have been successful in significantly increasing physical activity in college students [18,19,22,23]. SMS text messaging requires short messages and, therefore, education and graphics are limited. Facebook has also been used to encourage weight loss in college students [17,24]. Facebook allows for graphics and social networking but control of privacy may be an issue for users. Weight loss programs employing other Internet technology have been used successfully in overweight and obese college students [20,21]. Previous attempts to prevent excess weight gain by college students using eHealth have had mixed results [13,25-28]. Green [26] and Kattelmann [28] were able to demonstrate improvements in diet and physical activity behaviors, but no difference in weight gain or body measures, while Gow [25] and Levitsky [27] demonstrated the prevention of increases in body mass.

The objective of this pilot study was to determine to what extent a 24-week email-delivered health intervention program, which was designed to promote healthy diet and physical activity habits in college students, would be received by a diverse sample of college students and to report preliminary efficacy for diet, physical activity, and body measures. In terms of feasibility, we hypothesized that the enrollment goal (135 college students) would be met within the first 6 weeks of the semester and that > 60% of students enrolled would report reading at least 50% of the emails. Moreover, it was expected that after 24 weeks, those who received the eHealth intervention would consume more fruits and vegetables, less sugar and fat, and would report more physical activity than those in the control group. Furthermore, changes in diet and physical activity were anticipated to result in attenuation of body composition measures and improved levels of fitness.

## Methods

### Participants

Approval was obtained through the University Institutional Review Board (IRB). Eligible students were recruited from a large eastern university campus utilizing IRB-approved announcements via campus list-serve technology. Interested students contacted the study team for an initial phone screening to determine eligibility. Eligible students were then scheduled for an in-person informed consent visit. Students were eligible if they were enrolled in the university during the semester the study was conducted, were 18-20 years old, and had access to email. Informed consent was conducted by trained research staff at the first study visit, prior to any study procedures. Students were excluded if they were pregnant, lactating, reported a history of an eating disorder or bariatric surgery, were currently following a diet treatment plan for weight loss, or were participating in a research study that affected health behaviors. Eligible participants were randomized 2:1 and stratified by body mass index (BMI) and gender to the intervention or control group. This ratio ensured that dropouts did not affect the ability to demonstrate the feasibility of this pilot program [29].

Numbering of study weeks was linked to the baseline visit when the individual participant was randomized and began the electronic intervention, rather than on the academic calendar. Study enrollment was staggered over the first 6 weeks of the fall semester. The schedule for subsequent study visits was staggered similarly, to coincide with the follow-up surveys included in the intervention program. Participants were given a $25 gift card to a local department store for completing baseline, week 12, and week 24 study visits. Students who completed all visits were entered into a raffle for a $250 gift certificate.

### Anthropometric Measures

Trained research assistants conducted bioelectrical impedance analysis, anthropometric, and blood pressure measurements. All anthropometric data were performed in triplicate on the right side. Students were asked to refrain from exercise for 2 h, and maintain a 4-h fast (2 h for water, 12 h for caffeine and alcohol) prior to anthropometric measurements. A digital scale (Scale-Tronix, serial number 5002-27460) was used to weigh participants in underclothes to the nearest 0.1 kg. A wooden portable stadiometer (Shorr Productions) was used to measure participants’ height to the nearest millimeter, with the posterior heel, buttocks, shoulder blades, and head touching the vertical board of the stadiometer with the head in the Frankfurt plane [30]. The accuracy and precision of the scale and stadiometer were verified daily using two 20 kg weights and a 160 cm rod, respectively. A Gulick II fiberglass tape measure (Country Technology, model 67020) was used to measure body circumference to the nearest millimeter. Mid-neck circumference (NC) was measured between mid-cervical spine and mid-anterior neck just below the laryngeal prominence with the head in the Frankfurt plane [31]. Waist was measured at the right superior iliac crest [30]. Hip circumference was measured at the widest area across the buttocks [32]. Waist-to-hip ratio (WHR) was calculated by dividing the waist circumference (WC) measure in cm by the hip circumference measure in cm.

In preparation for bioelectrical impedance analysis (BIA), participants were questioned about fasting status, and were asked to empty their bladder. Urine samples were collected for pregnancy tests. All external metal from the right side of the body and any personal electronic devices were removed, to avoid interference in the flow of the electrical charge from the BIA. Pregnancy, history of seizures, and heart arrhythmias were considered exclusion criteria for BIA. Two participants were deferred, one for history of arrhythmias and a second for history of seizure. A third student failed to meet fasting conditions and did not complete baseline BIA. Participants rested supine avoiding skin-to-skin contact during the entire procedure. BIA was conducted according to the manufacturer’s instructions using an ImpediMed DF50 bioelectrical impedance analyzer. Briefly, skin of the right hand, wrist, foot, and ankle were scrubbed with alcohol wipes prior to placing 4 electrodes (1) midway between the distal ulna and the dorsal radius of the wrist; (2) 5 cm from the wrist electrode, toward the fingers; 3) midway between the distal tibia and dorsal fibula; and 4) 5 cm from the ankle electrode, toward the toes. Participant height, weight, sex, and age were entered into the software before conducting the BIA. Estimates of body composition were obtained from the BIA software.

### Diet and Physical Fitness

Students completed demographic, psychosocial, diet, and physical activity surveys online. The diet survey consisted of a food frequency questionnaire adapted from the Block Food Frequency Questionnaire [33]. The physical activity questionnaire was adapted from the Cross-Cultural Activity Patterns Questionnaire (CCAPQ) [34]. Reliability and validity have been established for both [34,35]. Exercise was defined as moderate to vigorous physical activity as categorized in the CCAPQ [36].

To prevent adverse reactions from fasting, each participant was offered water and snacks prior to the Queen’s College Step Test [37]. Snacks included fruit (6 types), cookies (4 types), and crackers and chips (11 types). Consumption of the snack was used to evaluate whether the eHealth messages resulted in actual diet change. Snacks were measured before serving to participants and again after the study visit. Postweight was subtracted from preweight to obtain total intake. Because consumption of snack was optional, frequency of consumption was used (the number of times students ate >50% of a snack divided by total snacks chosen at that visit) for each treatment group.

Seated blood pressure was measured under resting conditions using a 10 series automated blood pressure monitor (Omeron Healthcare) per the manufacturer’s instructions.

The Queen’s College Step Test procedure required participants to step up and down on a 41.3 cm wooden step for 3 min at 24 steps/min for males and 22 steps/min for females [37] while wearing an Omron HR-100C heart rate monitor transmitter. Step cadence was set using a metronome (Korg, model MA-30) and monitored using a Survivor III stopwatch (Accusplit, model S3MAGXLBK). Maximum oxygen utilization (VO_2max_) in mL/kg/min, was estimated for men (VO_2max_= 111.33 − 0.42 × heart rate in bpm) and women (VO_2max_= 65.81 − 0.1847 × heart rate in bpm) [37]. Two intervention group members were deferred from the Queen’s College Step Test due to uncontrolled asthma and injury.

### Intervention

The eHealth intervention consisted of A Lifestyle Intervention via Email (ALIVE), an evidence-based Web-based behavior change program created and managed by NutritionQuest [38] and modified by the authors for use with college students. Modifications primarily eliminated or replaced work and family-oriented language. For example, references to work and family were rewritten to include campus or apartment living and dining. Social and cognitive principles inherent to ALIVE included goal setting, a focus on individual choice, direct information and goal relevance for each learner, overcoming barriers, specific action-based advice such as establishing walking groups, salience of cues, building on prior learning, repetition of core messages, and repeated practice of new behaviors to transform them into sustained habits. There was an emphasis on small, achievable, and cumulative goals, for which accomplishment builds the participant’s self-efficacy to make changes and can enhance long-term maintenance.

After completing diet, physical activity, medical history, and stages of change surveys, participants received feedback comparing their responses to recommended levels of (1) fat and sugar intake, (2) fruit and vegetable intake, and (3) physical activity. As part of the ALIVE program, all participants were encouraged to select a goal related to the 3 feedback topics. Once randomized, participants in the intervention group chose one of these topics as the focus for weekly messages offering tailored small-step goals, tips for overcoming barriers to goals, health information, and social support*.* Web links in the intervention email also led students to their personal account on the ALIVE website, where educational information and feedback on progress were offered. Follow-up diet and physical activity surveys were delivered to participants every 12 weeks as part of ALIVE. A 24-week duration was chosen to best coincide with the school year. The intervention group received the adapted ALIVE program via weekly emails. The control group received weekly information related to nondiet, nonexercise health topics, such as distracted driving, sleep hygiene, and smoking cessation.

### Data Collection

Study data were collected and managed using Research Electronic Data Capture (REDCap) hosted at Children’s National Medical Center. REDCap is a secure, Web-based application that provides (1) an interface for validated data entry, (2) audit trails for tracking data manipulation and export procedures, (3) automated export procedures for data downloads to common statistical packages, and (4) procedures for importing data from external sources [39].

### Data Analysis

Demographic and baseline characteristics of students in the intervention group were compared to those in the control group using *t* test for continuous variables and chi-square for categorical variables. Chi-square was also used to determine differences in snack choice categories between the intervention and control groups. Changes over time were analyzed by repeated measures analysis of variance using a 2 (treatment: intervention, control) × 3 (time: baseline, week 12, week 24) factorial treatment design and sex and baseline BMI were controlled as covariates. Goodness-of-fit statistics were used to select the repeated measures structure that best fit the data. Significance was set at *P*<.05. The primary analysis was an intent-to-treat approach (ITT) with the last observation carried forward to fill in missing data. Secondary analyses were performed on a subgroup of completers. These participants were compliant with inclusion/exclusion criteria throughout the study period and completed all study visits and questionnaires.

## Results

### Participants

**Figure 1:**
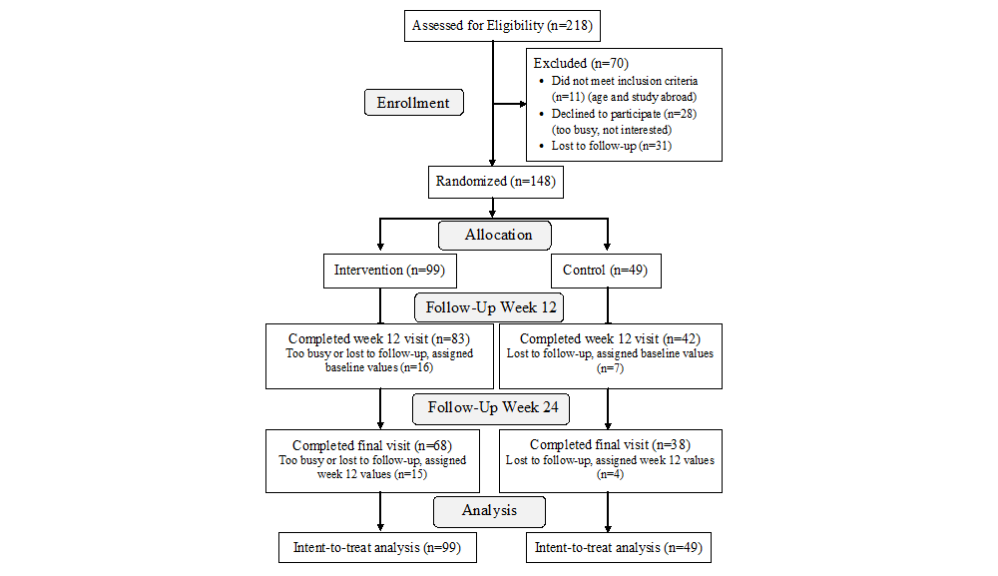
Consort flow diagram for college students recruited and retained.

An IRB-approved recruitment email was sent to 10,370 students, 693 of whom responded. The recruitment goal for this pilot study was 135 to retain 100 students with an expected dropout rate of 25%. The study team was able to contact 317 students by the last enrollment day; 99 did not respond and 218 were screened for eligibility, resulting in 99 students randomized to the intervention group and 49 to the control group (Figure 1). Mean age (standard error [SE]) was 19.7 (SE 0.06) years. Study participants were primarily female and racially diverse (Table 1). The participants were generally healthy, reporting low rates (1-5%) of hypertension, hypercholesterolemia, gastrointestinal disease, and anemia. The highest prevalence of disease was asthma (17%). Dietary supplement use was reported by 31% of students. Less than 5% of students were smokers. Other than previous research experience, baseline characteristics did not differ between treatment groups (Table 1).

Students who dropped out (intervention group: n=31, control group: n=11) had significantly higher mean BMI (24.2 [SE 0.62] kg/m^2^, *P*=.008), WC (85.3 [SE 1.55] cm, *P*=.002), WHR (0.86 [SE 0.0067] cm, *P*=.004), and NC (34.6 [SE 0.50] cm, *P*=0.006), yet lower energy intake (19.8 [SE 1.31] kcal/kg, *P*=.011), than those who completed all visits (BMI: 22.4 [SE 0.32] kg/m^2^, WC: 79.7 [SE 0.79] cm, WHR: 0.84 [SE 0.0043] cm, NC: 33.0 [SE 0.29] cm, and energy intake: 24.0 [SE 1.04] kcal/kg).

**Table 1:** Baseline characteristics of college students by treatment group

Characteristic		Intervention n (%)^a^	Control n (%)^a^	*P* ^b^
**Age (years): mean (SE^c^)**		19.8 (0.07)	19.6 (0.1)	.15
**Sex**				.93
	Female	68 (69)	33 (67)	
	Male	31 (31)	16 (33)	
**Race/ethnicity**				.64
	White, non-Hispanic	47 (47)	21 (43)	
	Asian	23 (23)	11 (22)	
	African-American	16 (16)	13 (26)	
	Hispanic	7 (7)	1 (2)	
	Mixed	4 (4)	2 (4)	
	Other	2 (2)	1 (2)	
**School year**				.15
	Freshman	3 (3)	5 (10)	
	Sophomore	38 (39)	23 (48)	
	Junior	53 (55)	19 (40)	
	Senior	3 (3)	1 (2)	
**Transportation to campus**				.55
	Live on campus	45 (46)	28 (57)	
	Walk/run	28 (29)	11 (22)	
	Drive	17 (17)	8 (16)	
	Bike	8 (8)	2 (4)	
Smoke		5 (5)	2 (4)	.76
Take vitamins		33 (34)	14 (29)	.58
Past research participant		48 (50)	10 (20)	.001

^a^Data are presented as total count and percent (intervention: N=99, control: N=49) unless otherwise noted. School year N=97 intervention, N=48 control. Transportation to campus N=98 intervention. Take vitamins: N=48 for control group. Past research participant: N=96 for intervention group.

^b^Student's t-test for age; all others are chi-square analysis of differences between groups.

^c^SE: standard error of the mean

### Anthropometrics

At baseline, 22% (33/148) were overweight/obese (BMI ≥25 mg/kg^2^). No significant differences were detected between treatment groups for students meeting health guidelines at baseline (Table 2). Baseline anthropometric data did not differ significantly between groups (Table 3). With the exception of WHR, most met recommendations for body measures. Further analysis of the WHR data revealed that all of those with elevated WHR were female, and of the 83 females with elevated WHR, 25 had WC measures that were below the recommended cut-off for disease risk (≤88 cm). Mean body fat mass percent (FM%) by sex was 29 in females and 20 in males.

### Diet and Fitness

At baseline, 88% of participants (130/148) reported consuming <5 servings of fruits and vegetables daily, 59% (87/148) consumed >10% of their kcal from saturated fat; however, 91% (135/148) met/exceeded 150 min/week of moderate-vigorous exercise. Prevalence of measured high blood pressure (18%, 26/148) was higher than self-reported blood pressure (0.7%, 1/148). No significant differences were detected between treatment groups for students meeting diet and physical fitness guidelines at baseline (Table 2).

**Table 2:** Percentage of college students meeting health recommendations at baseline.

Recommendation		Intervention n (%)^a^	Control n (%)^a^	*P* ^b^
**Anthropometrics**				
	FM% ^c^ (female: <32, male: <22)	54 (55)	33 (66)	.30
	BMI^d^ (18.8-25g/m^2^)	75 (76)	40 (80)	.60
	Neck circumference (female: <34 cm, male: <37 cm)	75 (76)	37 (74)	.68
	Waist circumference (female: ≤88 cm, male: ≤102 cm)	79 (81)	46 (92)	.08
	Waist-to-Hip ratio (Female: <0.8, male: <1.0)	43 (44)	21 (43)	.91
**Dietary intake**				
	Saturated fat (<10% daily kcal)	38 (38)	23 (46)	.58
	Total fat (<30% daily kcal)	97 (98)	49 (98)	.99
	Sugar (<10% daily kcal)	56 (57)	25 (50)	.45
	Fruit/vegetable (>5 servings/day)	13 (13)	5 (10)	.93
**Fitness**				
	Blood pressure (<120/80 mmHg)	83 (84)	39 (78)	.35
	Exercise^e^ (≥150 min/week)	88 (89)	47 (94)	.31
	VO_2max_^f^ (mL/kg/min)	56 (58)	34 (69)	.17

^a^Data are presented as total count and percent (intervention: N=99, control: N=49) except FM% (Intervention: N=96) and VO2max (intervention: N=94). Dietary intake data and exercise are self-reported.

^b^Chi-square analysis of differences between groups (*P*<.05).

^c^FM%: body fat mass percent

^d^BMI: body mass index

^e^Exercise: min/week of moderate to vigorous physical activity

^f^VO_2max_: maximum oxygen utilization. Met: >35, >33, >45, and >42 mL/kg/min for 19-year-old female, 20-year-old female, 19-year-old male, and 20-year-old male, respectively [40].

**Figure 2:**
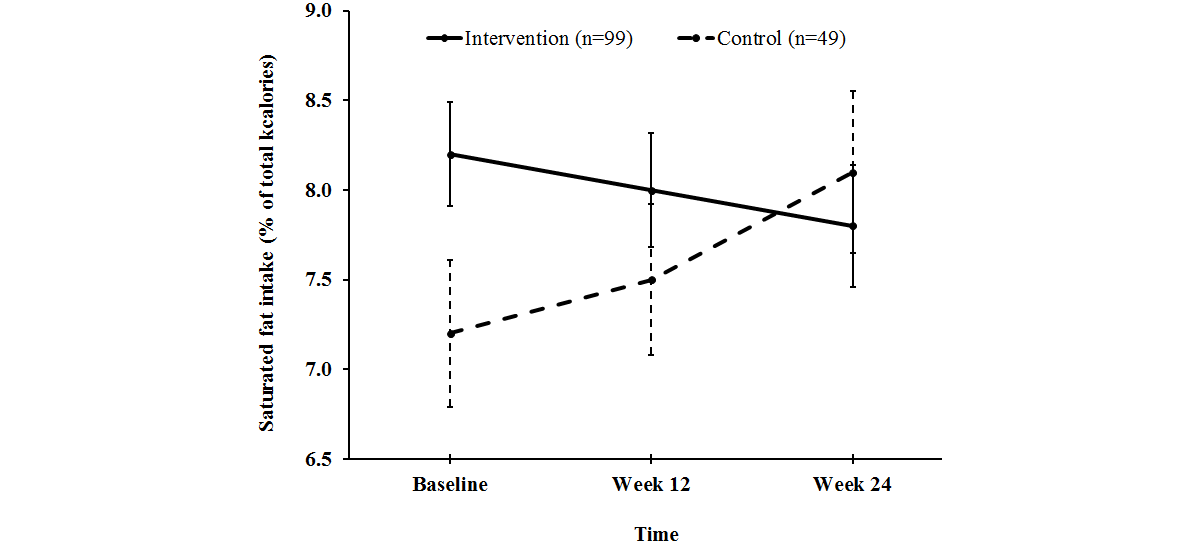
Saturated fat intake of college students over time by treatment presented as mean and standard error bars. Linear time by treatment interaction was significant (P = .048).

**Figure 3:**
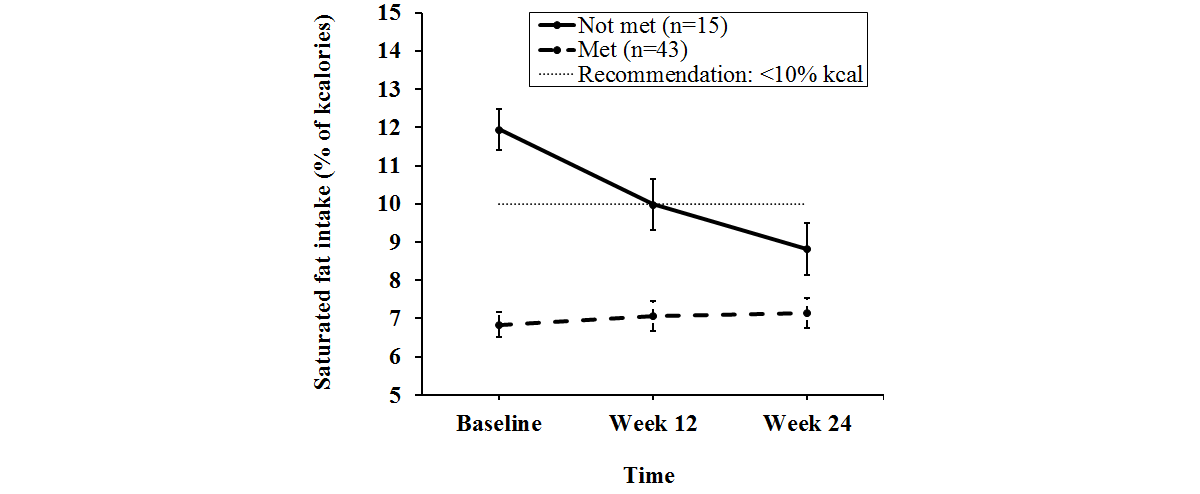
Change in saturated fat intake by college students in the intervention group who completed all study visits, categorized by met/not met recommendation at baseline, presented as mean and standard error bars. Slope of not met was significantly different than slope of met (P <.001).

**Figure 4:**
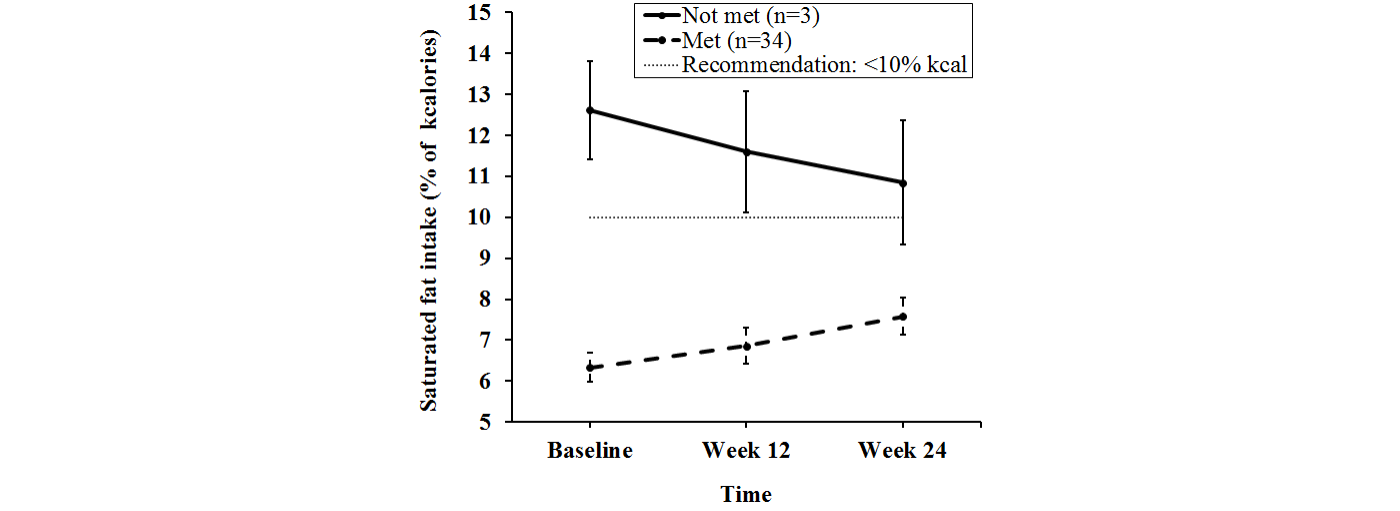
Change in saturated fat intake by college students in the control group who completed all study requirements, categorized by met/not met recommendation, presented as mean and standard error bars.

Repeated measures ANOVA with BMI and sex as covariates revealed a marginally significant linear time by treatment interaction for percent of energy from saturated fat, *F*_1,372_=3.94, *P*=.048 (Figure 2). This was true for ITT analysis and completers. This indicates that the increase in saturated fat intake by the control group (0.3 [SE 0.30] % of kcal) was different from the decrease in saturated fat intake by the intervention group (0.7 [SE 0.42] % of kcal), *P*=.048. No significant effect of BMI or sex was detected for these variables. Further analysis of the change in saturated fat intake according to the Dietary Guidelines for Americans (DGA) recommendation (<10% of kcal) revealed a significant linear interaction of time and meeting/not meeting recommendations for the intervention group (*P*<.001). Thus, the decrease in saturated fat intake in the intervention group was largely due to those whose intake was higher than recommended (Figure 3). Additionally, the mean saturated fat intake for completers in the intervention group did not meet the DGA guidelines at baseline but did reach DGA levels by study completion. In contrast, the three completers in the control group who consumed saturated fat in excess of DGA guidelines at baseline never achieved DGA levels (Figure 4). No significant treatment by time interactions were detected for dietary intake of sugar, or fruit and vegetables, or for anthropometric or fitness variables (Table 3).

**Figure 5:**
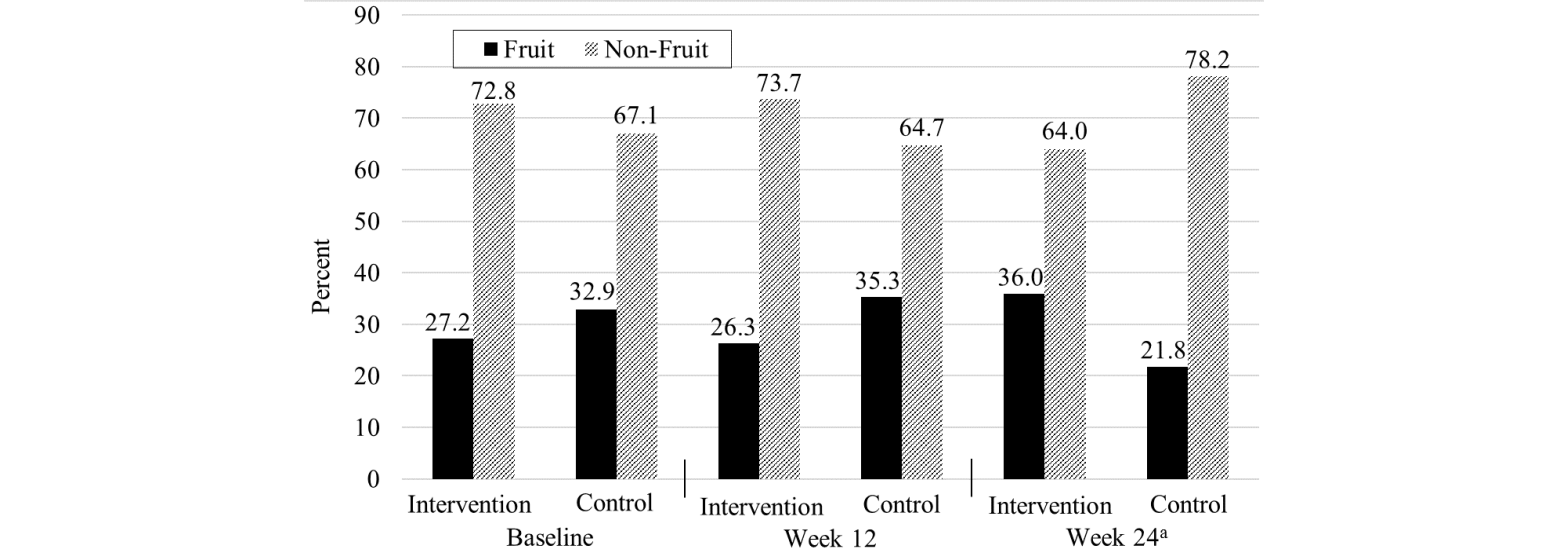
Percentage of fruit and non-fruit snacks consumed by college students. Significant difference was observed between treatment groups at week 24 only (P < .001).

No significant difference in consumption of fruit or non-fruit snacks at baseline (*P*=.22) and week 12 visits (*P*=.06) was detected between the intervention and control groups. At week 24, significantly different consumption of fruit and non-fruit snacks (χ^2^_1, N=221_ = 11.7, *P*<.001) was detected between the intervention and control groups (Figure 5).

**Table 3:** Body measures, dietary intake, and physical fitness of college students over time by treatment.

Variable		Baseline^a^		Week 12^a^		Week 24^a^		
		Int	Cont	Int	Cont	Int	Cont	*P*
**Anthropometrics**								
	FM%^b^	24.3 (0.50)	25.0 (0.68)	24.9 (0.50)	25.2 (0.68)	24.6 (0.50)	24.9 (0.68)	0.36
	BMI^c^ (kg/m^2^)	23.1 (0.38)	22.8 (0.54)	23.1 (0.38)	22.8 (0.54)	23.2 (0.38)	22.8 (0.54)	0.80
	NC^d^ (cm)	34.4 (0.14)	34.4 (0.19)	34.4 (0.14)	34.4 (0.19)	34.5 (0.14)	34.6 (0.19)	0.34
	WC^e^ (cm)	82.2 (0.49)	79.9 (0.68)	81.7 (0.49)	80.4 (0.68)	81.9 (0.54)	80.1 (0.75)	0.41
	WHR^f^	0.85 (0.004)	0.84 (0.006)	0.85 (0.004)	0.84 (0.005)	0.85 (0.004)	0.84 (0.006)	0.21
**Dietary intake**								
	Saturated fat^g^ (% total kcal)	8.2 (0.29)	7.2 (0.41)	8.0 (0.32)	7.5 (0.42)	7.8 (0.34)	8.1 (0.45)	0.14
	Sugar (% total kcal)	10.6 (0.84)	12.1 (1.2)	10.2 (0.84)	12.0 (1.1)	10.1 (0.84)	10.6 (1.2)	0.32
	Fruit/veg^h^ (cups/day)	2.6 (0.17)	2.7 (0.23)	2.4 (0.14)	2.3 (0.20)	2.4 (0.14)	2.4 (0.20)	0.64
**Fitness**								
	SBP^i^ (mmHg)	111 (0.72)	111 (1.2)	111 (0.89)	111 (1.2)	110 (0.89)	110 (1.2)	0.92
	DBP^j^ (mm Hg)	71.9 (0.72)	70.8 (1.0)	71.8 (0.72)	70.7 (1.0)	71.0 (0.72)	70.2 (1.0)	0.80
	Exercise^k^ (min/week)	795 (58)	844 (80)	736 (58)	705 (80)	788 (58)	801 (80)	0.63
	VO_2max_^l^ (mL/kg/min)	41.4 (0.59)	42.6 (0.81)	41.1 (0.44)	42.1 (0.59)	40.8 (0.47)	42.4 (0.63)	0.83

^a^Int = Intervention. Cont = Control. Overall N=99 Int, N=49 Cont except FM% N=96 for Int, and VO2max N=94 for Int. Mean (standard error) derived by ANOVA repeated measures intent-to-treat analysis, adjusted for sex and baseline BMI.

^b^FM%: Fat Mass%

^c^BMI: body mass index

^d^NC: neck circumference

^e^WC: waist circumference

^f^WHR: waist-to-hip ratio

^g^Linear time by treatment interaction: F_1,372_=3.94, *P*=.048

^h^Fruit/veg: fruit and vegetable

^i^SBP: systolic blood pressure

^j^DBP: diastolic blood pressure

^k^Exercise: minutes per week of reported moderate-vigorous activity

^l^VO_2max_: maximum oxygen utilization

### Intervention

Among the 99 students in the intervention group, 32 chose goals to reduce fat and sugar intake, 32 chose to improve fruit and vegetable intake, and 35 chose to focus on physical activity. Of the 109 students who answered the satisfaction survey at week 24, 91 (84%) reported reading at least half of the study emails (control: n=35, intervention: n=56).

## Discussion

### Principal Findings

This study demonstrated the feasibility of ALIVE in a college population, by exceeding the recruitment goal with 106 participants who completed the study, a majority of whom reported reading most of the emails. ALIVE was effective in decreasing consumption of saturated fat and increasing the frequency of choosing fruit as a snack by a diverse sample of college students.

### Intervention

Similar to other eHealth interventions in college students, small improvements in dietary intake but no changes in body measures were seen in the current study [26,28]. Differences between treatment groups in dietary intake of sugar, energy, vegetables, or for anthropometric or fitness variables were not detected. However, attenuation of BMI, WC, NC, WHR, body fat, blood pressure, sugar intake, and VO_2max_ in this study indicated that student health habits had not worsened. As seen in Table 2, large percentages of students required improvements in dietary sugar, fruit/vegetable, and saturated fat intake, whereas most students met requirements for body measures and physical activity. Additional studies are needed to assess the efficacy of eHealth in reinforcing optimal behaviors, particularly for those who do not meet health recommendations.

That the intervention group was not more successful compared to the control in attenuating health habits may arise from the insufficient power to detect these treatment effects, and the bias of repeated diet and physical activity surveys and body measurements in both treatment groups. According to previous studies, college students who are measured but receive no feedback, education, or intervention, show an increase in BMI, FM%, WC, and dietary fat intake, and decreased vegetable and fruit intake over time [9,10,41]. In the present study, control participants also received feedback and were encouraged to select a goal at baseline. Additionally, students in the intervention group were able to switch between the fruit and vegetable, fat and sugar, and physical activity health behavior tracks at any time. Thus, it is likely that only some students had the benefit of the full program of coaching. This could have weakened the effect, requiring a much larger sample size. It should also be noted that week 12 visits coincided with fall semester finals, which can be a high-snack, low-activity period in college life.

### Anthropometrics

Although some eHealth programs were able to prevent increases in weight gain by college students, we were not able to support this finding [25,27]. However, the current study did demonstrate the importance of screening for high blood pressure in college populations and the importance of carefully selecting anthropometric measurements in order to detect disease risk. This study is the first to report NC in a diverse college sample [41,42]. A relatively unused measure, NC has been associated with obesity and cardiovascular disease risk [31,43]. In the current sample, approximately 25% of students were categorized as obese using NC, which is comparable to that identified by BMI. WC and WHR are often used to assess central obesity and are predictive of insulin resistance [44] and cardiovascular disease [41,45,46]. In our sample, 30% of females with elevated WHR had WC measures in the healthy range, indicating that WHR may not be a reliable indicator of central adiposity in a college-aged sample. NC is less intrusive than WC and should be further explored as an indicator of adiposity and disease risk in college students.

The students who failed to complete the study had significantly higher indicators of overweight/obesity, although only NC was clinically significant (higher and over recommendation) compared to those who dropped out. This may indicate that the program may not be well received by those with higher body weight.

### Diet and Fitness

Less than half of the participants met the dietary guidelines for saturated fat intake at baseline, whereas the amount of total fat consumed met recommended levels. Thus, college students could benefit from interventions that alter food choices to improve the fat profile. The ALIVE program used in this study was designed to encourage and measure dietary changes in fat, sugar, fruits, and vegetables. We detected a linear time by treatment interaction, in which saturated fat intake worsened for the control group compared to the intervention group. Considering that the dietary guideline is 10% or less of calories from saturated fat [47], the observed difference in intake between groups of 1% of calories is clinically important.

Only 6% of college students report eating at least five servings of fruits and vegetables daily [14]. This is further supported by the current study, in which less than 15% of participants met the dietary guideline for fruit and vegetables. Snacks are a convenient way to bolster fruit and vegetable intake. Many are “self-packaged” and easy to carry (eg, oranges, apples, bananas). Given the limits of self-reported data, consumption of fruit as a snack was objectively measured in this sample of college students. Improved selection and consumption of fruit when offered as a snack was found after 24 weeks of the ALIVE program, whereas self-reported data showed no change in fruit and vegetable intake.

Lack of fitness as indicated by VO_2max_ and poor dietary patterns may explain the large portion of students having excessive weight and body fat. For example, almost half of the students in our study exceeded the World Health Organization recommendation for sugar intake, while a majority of students did not meet DGA guidelines for consuming fruit and vegetables. Dietary intake in this sample is consistent with body measures that indicate excessive weight in about a quarter of students studied.

In the current study, physical fitness was assessed by the Queen’s step test, which estimates VO_2max_. Categories of physical fitness as determined by VO_2max_ have been established by sex and age [40]. Higher numbers for VO_2max_ indicate better physical fitness. Overall, mean VO_2max_ placed the student sample in the “good” category for physical fitness level at baseline. Sixty-two percent of students had optimal physical fitness, which differs from the survey findings in which 90% of students reported engaging in the recommended 150 min/week of moderate to vigorous physical activity. Overreporting may explain the difference between the survey results and the fitness test results.

### Limitations

As with most health behavior studies, self-selection bias of the convenience sample was a limitation in this study. Students who are interested in health are motivated to participate and may be more likely to improve health habits than others. The additional research experience in the intervention group could also have contributed to reporting bias between treatment groups.

Another major limitation involved exposure to feedback and promotion of health goals in the control group after the initial diet and physical activity surveys. Future studies could include a waitlist control or manipulate the initial survey to exclude feedback and goal selection.

Anthropometric measurements may be subject to some amount of error. For example, menstrual cycles may affect body measurements. We did not account for these potential confounding variables during the study.

Reported intake is known to be biased, with underreporting directly associated with BMI [48,49]. ALIVE used food frequency questionnaires to identify intake. Food frequency questionnaires are more biased and less correlated than food diaries [49]. Actual measurement of food intake is costly and time consuming. This study included the novel method of exploring the use of objectively measured partial dietary intake by measuring snack intake at study visits.

The fact that participants could choose very different goals (ie, increasing fruit/vegetable intake, decreasing fat/sugar intake, or increasing physical activity) and could switch tracks during the program indicated that we did not have sufficient power. Goal switching as a limitation has been addressed in ALIVE-PD, a future version of ALIVE, which is currently being evaluated for efficacy in adults. In ALIVE-PD, users work on diet and physical activity goals simultaneously, rather than switching between goals. Larger studies using ALIVE-PD in college students are warranted to document changes in diet and physical activity.

### Conclusions

ALIVE is feasible in a diverse college population, as demonstrated by high participation and retention. Furthermore, health behaviors of college students, such as reducing saturated fat intake and increasing fruit intake can be influenced by ALIVE. Large randomized control trials using adaptations of ALIVE are warranted.

## Abbreviations

ALIVE: A Lifestyle Intervention Via Email
BIA: bioelectrical impedance analysis
BMI: body mass index
CCAPQ: Cross-Cultural Activity Patterns Questionnaire
DBP: diastolic blood pressure
DGA: Dietary Guidelines for Americans
eHealth: electronic health interventions
FM%: body fat mass percent
IRB: Institutional Review Board
ITT: intent-to-treat
NC: neck circumference
REDCap: Research Electronic Data Capture
SBP: systolic blood pressure
VO2max: maximum oxygen utilization
WC: waist circumference
WHR: waist-to-hip ratio
